# Bayesian estimation of the number of protonation sites for urinary metabolites from NMR spectroscopic data

**DOI:** 10.1007/s11306-018-1351-y

**Published:** 2018-03-26

**Authors:** Lifeng Ye, Maria De Iorio, Timothy M. D. Ebbels

**Affiliations:** 1 Department of Statistical Science, University College London, London, UK; 2 Department of Surgery and Cancer, Computational and Systems Medicine, Imperial College London, London, UK

**Keywords:** NMR, pH, Peak shift changes, Protonation site, Bayesian model selection

## Abstract

**Introduction:**

To aid the development of better algorithms for $^1$H NMR data analysis, such as alignment or peak-fitting, it is important to characterise and model chemical shift changes caused by variation in pH. The number of protonation sites, a key parameter in the theoretical relationship between pH and chemical shift, is traditionally estimated from the molecular structure, which is often unknown in untargeted metabolomics applications.

**Objective:**

We aim to use observed NMR chemical shift titration data to estimate the number of protonation sites for a range of urinary metabolites.

**Methods:**

A pool of urine from healthy subjects was titrated in the range pH 2–12, standard $^1$H NMR spectra were acquired and positions of 51 peaks (corresponding to 32 identified metabolites) were recorded. A theoretical model of chemical shift was fit to the data using a Bayesian statistical framework, using model selection procedures in a Markov Chain Monte Carlo algorithm to estimate the number of protonation sites for each molecule.

**Results:**

The estimated number of protonation sites was found to be correct for 41 out of 51 peaks. In some cases, the number of sites was incorrectly estimated, due to very close pKa values or a limited amount of data in the required pH range.

**Conclusions:**

Given appropriate data, it is possible to estimate the number of protonation sites for many metabolites typically observed in $^1$H NMR metabolomics without knowledge of the molecular structure. This approach may be a valuable resource for the development of future automated metabolite alignment, annotation and peak fitting algorithms.

## Introduction

$^1$H NMR is an important technique in metabolomics as it provides highly reproducible, quantitative information on a wide variety of metabolites. The chemical shift and multiplicity pattern are characteristic of the metabolite’s chemical structure, but are complicated by small sample-to-sample changes in the position of individual resonances due to changes in pH, ionic strength or other physical parameters of the matrix (Fan 1996). While these can be ameliorated to some degree by careful analytical procedures, such as addition of buffers and control of physical conditions, changes in chemical shifts are still present in most NMR metabolomic data sets. Computational approaches to correct these changes, such as alignment, can introduce artefacts and are not able to correct shift changes which swap the ordering of resonances (Vu and Laukens 2013). Chemical shift changes can become a major problem in the statistical analysis of NMR metabolomics data, as they disrupt the linear relationship between NMR intensity at a given position and metabolite abundance (Ebbels and Cavill 2009). Thus it becomes important to characterise and model chemical shift changes (see e.g. Takis et al. 2017), in part to aid construction of better algorithms for data analysis, such as alignment or peak-fitting. We recently reported titration model parameters such as acid/base limits and pKas for 33 identified metabolites in human urine, as well as titration curves for a further 65 unidentified peaks (Tredwell et al. 2016). A key problem in modelling NMR spectra from untargeted metabolomics is the unknown structure of the molecules giving rise to each resonance, and thus the lack of knowledge of important parameters. In particular, the number of proton binding sites strongly influences relationship of chemical shift to pH, but has traditionally been hard to infer from titration data alone. Here, we report the successful development and application of a Bayesian approach to estimating the number of proton binding sites in $^1$H NMR metabolomics data, without knowledge of the molecule’s chemical structure.

## Methods

### The model

As protonation is usually rapid and reversible on the NMR timescale, the theoretical chemical shift ($\tilde{\delta }$) is a weighted average of the limiting chemical shifts of the unprotonated ($\delta _{A}$) and the protonated ($\delta _{HA}$) states of the molecule (Ackerman et al. 1996; Szakács et al. 2004). Ackerman et al. (1996) model the theoretical chemical shift as a function of pH and pKa as follows

1 $$\begin{aligned} \tilde{\delta } = \frac{\delta _A + \delta _{HA}(10^{(\text{pH}-\text{pKa})})}{1+10^{(\text{pH}-\text{pKa})}} \end{aligned}$$

Szakács et al. (2004) extend this approach to molecules with $q>1$ protonation sites:

2 $$\begin{aligned} \tilde{\delta }= \frac{\delta _A+\sum ^{q}_{i=1}\delta _{HiA}10^{\left(\sum ^q_{j=q-i+1}pK_j\right)-i\text{pH}}}{1+\sum ^q_{k=1}10^{\left(\sum ^q_{l=q-k+1}pK_l\right)-k\text{pH}}}, \end{aligned}$$

accounting for the interaction between protons bound at different binding sites and the statistics of proton binding.

From Eqs. (1, 2), it is evident that the theoretical chemical shift follows a titration curve which describes the position of the resonance over a range of pH. When the number of sites is known, nonlinear fitting can be applied using Eq. (2) to model the titration curve to obtain the pKa values, as well as the acid and base chemical shift limits (Tredwell et al. 2016). However, in many metabolomics applications (for example alignment), the number of protonation sites may not be known, especially for unknowns or molecules of complex structure. Thus it is of interest to consider whether the number of protonation sites can be estimated along with the pH dependence of the chemical shift.

Here, we focus on inferring the number of protonation sites from observations of chemical shift changes for a given resonance at different pH values. Due to their small size, few metabolites have many protonation sites. We therefore limit the search space to 1-site, 2-site and 3-site models, although the approach can be easily extended to include more than 3 protonation sites if required. We employ a Bayesian approach because it provides a natural way of incorporating prior information and combining results of different experiments. In the Bayesian framework, it is, in principle, easy to incorporate model choice in the inferential process by specifying an appropriate prior distribution on the model space. Posterior inference is performed through Markov chain Monte Carlo (MCMC) methods. In this context, as model selection involves models with different dimensions, we employ a Reversible jump MCMC algorithm, which is implemented in the software JAGS (Plummer and Martyn 2003).

We propose a non-linear Bayesian regression model for each NMR resonance for each molecule. In particular, we assume that the observed chemical shift, $y_i$, follows a normal distribution, with mean $\tilde{\delta }$, representing the theoretical chemical shift, and variance $\sigma ^2$, the measurement error:

$$\begin{aligned} y_i|\tilde{\delta }_i,\sigma ^2 \sim N(\tilde{\delta },\sigma ^2) \end{aligned}$$

The theoretical chemical shift $\tilde{\delta }_i$ is a function of the pH, pKa, $\delta _A$ and the number of protonation sites as described in Eq. (2).

### Specification of prior knowledge

Since most metabolites have up to three protonation sites, we specify as prior distribution on the number of protonation sites a uniform distribution on the set $\{1,2,3\}$. Therefore, each model is a priori equally likely. We complete the model by specifying a prior distribution on the remaining parameters. Assuming no additional spectral effects and conditioning on the number of sites *q*, we choose a uniform distribution defined over the NMR ppm scale [0, 10] as prior for $\delta _A$ and $\delta _{H_jA}, j=1,\ldots ,q$.

Moreover, to improve efficiency in searching the parameter space and avoid identifiability issues (where different combinations of parameter values lead to the same likelihood value so that the model is not able to distinguish between them) we impose an order constraint on the $\mathbf {\delta _A}$ and $\mathbf {\delta _{H_jA}}$ values, in descending or ascending order according to the trend of the data. This improves MCMC convergence and the accuracy of estimation. The order direction can be estimated, for example, by fitting a simple linear regression, $\mathbf {y} = \beta \mathbf{pH} + b$, to the data and considering the sign of the estimated slope parameter $\beta$. If $\beta> 0$, the relationship between chemical shift and pH is approximately increasing and we would impose the constraint $\delta _{A}> \delta _{{H_{1} A}}> \delta _{{H_{2} A}}> \cdots> \delta _{{H_{q} A}}$ on the parameter space. On the other hand, if $\beta<0$, we would impose restriction $\delta _{A} < \delta _{{H_{1} A}} < \delta _{{H_{2} A}} < \cdots < \delta _{{H_{q} A}}$.

For most metabolites the change in chemical shift between adjacent protonation sites is smaller than 1ppm and the total shift change from most acidic to most basic peak position is also smaller than 1ppm. This allows us to assume that the change of chemical shift between adjacent protonation sites is smaller than 1ppm, i.e.

$$\begin{aligned} 0<|\delta _A-\delta _{H_1A}|<1\;\;\;\;\;\;0<|\delta _{H_jA}-\delta _{H_{j+1}A}|<1,\;\; j =1,2,3,... \end{aligned}$$

Finally, an Inverse-Gamma prior distribution with parameters ($a^2,a$), which is often used as a Bayesian prior for error variance, is chosen for $\sigma ^2$. Note that *a*, which reflects the measurement error, should be chosen carefully according to the experiment. In our model, $a = 10^4$ is chosen based on empirical estimation of the measurement error related to the resolution of the spectrometer and its ability to measure peak position (Karakach et al. 2009).

We fit the model to each resonance independently. We pick as an estimate of *q* the number of protonation sites with highest posterior probability. We then refit the same model but fix *q* equal to its posterior estimate to obtain an estimate of the other parameters conditional on *q*. Posterior inference is performed in JAGS, running four chains of the MCMC algorithm for 50,000 iterations with a burn-in period of 25,000.

### Prior specification for pKa

A great advantage of working in a Bayesian framework is the ability of the model to incorporate problem specific prior information. To assign informative prior knowledge on the pKa range, which aids computational stability and improves convergence of the MCMC algorithm, we exploit information available in the the Human Metabolome Database (version 4.0), which records the pKa values of many common urine metabolites.

By studying the empirical distribution of the pKa values downloaded from the database, we found that the distribution of pKa values has a heavy right tail. We choose as prior range for pKa [1.2, 13.7] to correspond to the pH range of our data. This range includes most urine metabolites reported in HMDB, but excludes values below the 7% and above the 90% percentile of the pKa distribution.

### Data

Details of sample collection, NMR acquisition and data processing can be found in Tredwell et al. (2016). All data used in this study is publically available as Supplementary material to the original article under the Creative Commons attribution 4.0 International License https://creativecommons.org/licenses/by/4.0/. Briefly, a urine sample was collected from five different individuals and pooled to obtain an average representative human urine sample. To avoid chemical shift effects from metal ions the urine was treated with chelex resin to reduce both $\text {Ca}^{2+}$ and $\text {Mg}^{2+}$ concentrations without significantly altering metabolite composition. Note that, while this results in non-physiological concentrations of these ions, it is not expected to affect the ability of the model to recover the number of protonation sites. The pool was then titrated to produce 51 samples covering the range $2<\mathtt {pH}<12$. Spectra were acquired on a Bruker Avance DRX600 NMR spectrometer (Bruker BioSpin, Rheinstetten, Germany), with a $^1$H frequency of 600 MHz. A one-dimensional NOESY sequence was used with water suppression, and data were acquired into 64k data points over a spectral width of 12 KHz, with eight dummy scans and 64 scans per sample. Spectra were processed in iNMR 3.4 (Nucleomatica, Molfetta, Italy). Fourier transform of the free-induction decay was applied with a line broadening of 0.5 Hz. Spectra were manually phased and automated first order baseline correction was applied. Metabolites were assigned using the Chenomx NMR Suite 5.1 (Chenomx, Inc., Edmonton, Alberta, Canada) relative to 4,4-dimethyl-4-silapentane-1-sulfonic acid (DSS) as an internal standard. Metabolite peak positions were obtained using in-house MATLAB scripts. Data for one metabolite (phenylalanine at 7.35 and 7.41 ppm) were discarded as it was found that the peak positions could not be measured accurately due to the high level of peak overlap in this region of the spectra.

## Results and discussion

**Fig. 1:**
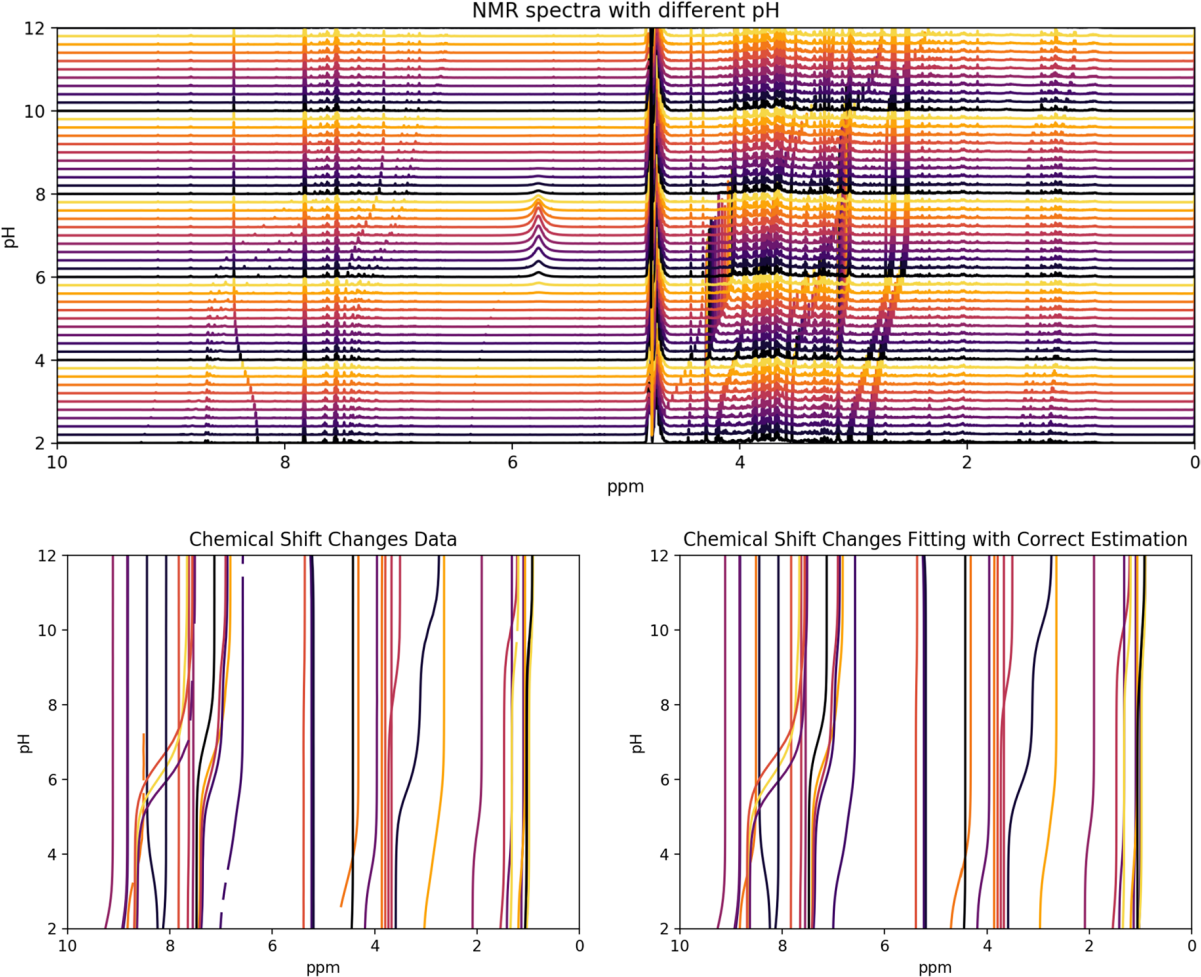
Upper panel: $^1$H NMR spectra with pH adjusted from 2 to 12. Lower left panel: observed chemical shift positions of 51 resonances. Lower right panel: fitted chemical shift positions for the 51 resonances. Only resonances with correct predictions are shown

Our aim is to estimate the number of protonation sites for small molecule metabolites from their observed NMR pH titration curves. From Fig. 1, it is clear that when the number of protonation sites is estimated correctly, the chemical shift changes match the data quite well.

A summary of the results is shown in Table 1. More detailed results for each resonance can be found in Table 2. Of the 51 resonances, the estimated number of sites matches that found in the literature in 41 cases ($80.4\%$). It is evident that most of the incorrect predictions (10 out of 51) result from an underestimation of the number of sites compared to the literature value. The literature site numbers are sourced from handbook of biochemistry and molecular biology (Lundblad and Macdonald 2010). Where this was not possible, (hydroxyisobutyrate, hydroxyisovalerate, indoxyl and methyl-2-oxovalerate) the number was determined from an assessment of the molecular structure.

**Table 1:** Comparison of the literature number of sites and the number estimated by the model

		Estimated number of sites			Total
		1	2	3	
Literature number of sites	1	**25**	1	0	26
	2	5	**9**	0	14
	3	0	4	**7**	11
Total		30	14	7	51

Correctly estimated numbers of sites are shown in bold

**Table 2:** Probability of different numbers of protonation sites, estimated number of protonation sites and literature number of protonation sites for 51 resonances from 32 metabolites in human urine

Metabolite	Database ID	Chemical shift at pH7.4	1 Site prob.	2 Site prob.	3 Site prob.	Estimated number of sites	Literature number of sites
**Hydroxyisobutyrate**	**HMDB0000729**	**1.347**	**91.893**	**7.488**	**0.619**	**1**	**1**
**Hydroxyisovalerate**	**HMDB0000754**	**1.260**	**86.597**	**12.795**	**0.608**	**1**	**1**
**Indoxyl**	**HMDB0004094**	**7.192**	**93.017**	**5.688**	**1.295**	**1**	**1**
**Methyl-2-oxovalerate**	**HMDB0000695**	**1.093**	**95.383**	**4.326**	**0.291**	**1**	**1**
**Acetate**	**HMDB0000042**	**1.910**	**93.326**	**5.879**	**0.795**	**1**	**1**
**Alanine**	**HMDB0000161**	**1.212**	**0**	**80.827**	**19.173**	**2**	**2**
**Allantoin**	**HMDB0000462**	**5.383**	**97.868**	**2.049**	**0.083**	**1**	**1**
Citrate	HMDB0000094	2.528	0	75.404	24.596	2	3
**Citrate**	**HMDB0000094**	**2.646**	**0**	**47.869**	**52.131**	**3**	**3**
Creatinine	HMDB0000562	3.033	87.889	11.593	0.518	1	2
Creatinine	HMDB0000562	4.043	94.992	4.65	0.358	1	2
**Formate**	**HMDB0000142**	**8.448**	**92.786**	**6.377**	**0.837**	**1**	**1**
**Glucose**	**HMDB0000122**	**5.228**	**98.661**	**1.284**	**0.055**	**1**	**1**
**Hippurate**	**HMDB0000714**	**3.960**	**70.111**	**27.403**	**2.486**	**1**	**1**
**Hippurate**	**HMDB0000714**	**7.541**	**86.036**	**9.798**	**4.166**	**1**	**1**
**Hippurate**	**HMDB0000714**	**7.627**	**92.742**	**6.114**	**1.144**	**1**	**1**
**Hippurate**	**HMDB0000714**	**7.824**	**92.264**	**5.387**	**2.349**	**1**	**1**
**Hippurate**	**HMDB0000714**	**8.512**	**54.082**	**26.841**	**19.077**	**1**	**1**
**Histidine**	**HMDB0000177**	**7.253**	**0**	**42.669**	**57.331**	**3**	**3**
**Histidine**	**HMDB0000177**	**8.188**	**0**	**7.55**	**92.45**	**3**	**3**
**Imidazole**	**HMDB0001525**	**7.229**	**59.201**	**37.952**	**2.847**	**1**	**1**
Imidazole	HMDB0001525	8.040	0	73.582	26.418	2	1
**Isoleucine**	**HMDB0000172**	**0.902**	**0.801**	**65.964**	**33.235**	**2**	**2**
**Lactate**	**HMDB0000190**	**1.320**	**89.155**	**9.941**	**0.904**	**1**	**1**
Leucine	HMDB0000687	0.932	83.263	14.099	2.638	1	2
**Mannitol**	**HMDB0000765**	**3.673**	**92.487**	**6.501**	**1.012**	**1**	**1**
**Mannitol**	**HMDB0000765**	**3.797**	**96.633**	**2.676**	**0.691**	**1**	**1**
**Mannitol**	**HMDB0000765**	**3.864**	**96.567**	**2.964**	**0.469**	**1**	**1**
**Methylsuccinate**	**HMDB0001844**	**1.062**	**43.561**	**50.274**	**6.165**	**2**	**2**
**Piperazine**	**HMDB0014730**	**3.526**	**0**	**65.255**	**34.745**	**2**	**2**
**TMethylHistidine**	**HMDB0000479**	**6.873**	**0**	**14.792**	**85.208**	**3**	**3**
**TMethylHistidine**	**HMDB0000479**	**8.306**	**0**	**0**	**100**	**3**	**3**
TTMethylHistidine	HMDB0000001	3.788	0	94.773	5.227	2	3
**TTMethylHistidine**	**HMDB0000001**	**6.909**	**0**	**16.988**	**83.012**	**3**	**3**
**TTMethylHistidine**	**HMDB0000001**	**8.396**	**0**	**23.514**	**76.486**	**3**	**3**
**Tartrate**	**HMDB0029878**	**4.322**	**5.764**	**51.864**	**42.372**	**2**	**2**
Taurine	HMDB0000251	3.412	86.863	7.137	6	1	2
**Threonine**	**HMDB0000167**	**1.194**	**14.472**	**67.882**	**17.646**	**2**	**2**
**Trigonelline**	**HMDB0000875**	**4.429**	**68.693**	**19.481**	**11.826**	**1**	**1**
**Trigonelline**	**HMDB0000875**	**8.073**	**74.875**	**17.025**	**8.1**	**1**	**1**
**Trigonelline**	**HMDB0000875**	**8.822**	**77.588**	**15.531**	**6.881**	**1**	**1**
**Trigonelline**	**HMDB0000875**	**8.834**	**58.857**	**30.807**	**10.336**	**1**	**1**
**Trigonelline**	**HMDB0000875**	**9.115**	**67.411**	**21.353**	**11.236**	**1**	**1**
**Tris**	**CHEBI:9754**	**3.715**	**94.453**	**5.363**	**0.184**	**1**	**1**
Tryptophan	HMDB0000929	7.719	87.38	11.156	1.464	1	2
Tyrosine	HMDB0000158	6.885	0	90.202	9.798	2	3
Tyrosine	HMDB0000158	7.207	0	90.592	9.408	2	3
**Valine**	**HMDB0000883**	**0.906**	**0**	**79.851**	**20.149**	**2**	**2**
**Valine**	**HMDB0000883**	**1.060**	**2.967**	**77.568**	**19.465**	**2**	**2**
**Xylose**	**HMDB0000098**	**5.190**	**98.475**	**1.476**	**0.049**	**1**	**1**
**Trans-aconitate**	**HMDB0000958**	**6.574**	**0**	**64.477**	**35.523**	**2**	**2**

Rows with correctly estimated numbers of sites are shown in bold

**Fig. 2:**
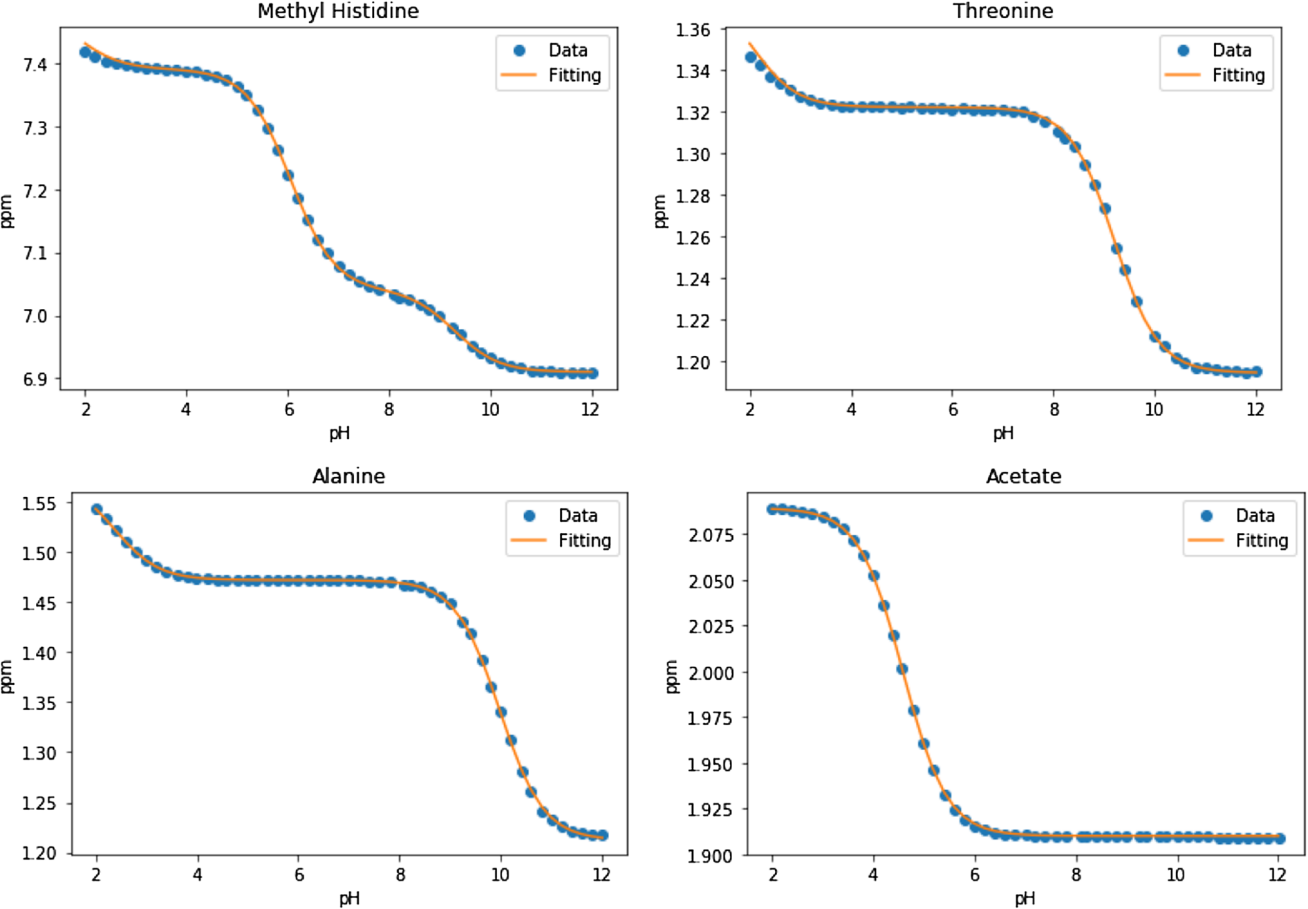
Measured chemical shift changes for acetate, alanine, threonine and TTMethylHistidine with the fit of the theoretical model

Given the estimation of the number of protonation sites, the other parameters of the model (acid limits, base limits and pKa values) can be estimated using the same model. The modelled pKa values closely agree with the literature values (Lundblad and Macdonald 2010), and the modelled acid and base limits are also in good agreement with the previously modelled values (Tredwell et al. 2016). Therefore we do not present these in detail here. Four examples including 1, 2 and 3 protonation sites, (acetate, alanine, threonine and TTMethylHistidine) are shown in Table 3 and Fig. 2.

**Table 3:** Literature and modelled results of acetate, alanine, threonine and TTMethylHistidine

Metabolite	Literature pKa values			Modelled pKa values			Modelled acid and base limits			
Acetate	4.760			4.591			1.910	2.089		
Alanine	2.340	9.690		2.384	9.980		1.212	1.472	1.573	
Threonine	2.630	10.430		2.072	9.195		1.194	1.322	1.379	
TTMethylHistidine	1.690	6.480	8.850	1.832	6.062	9.302	6.910	7.040	7.390	7.491

### Metabolites with incorrectly estimated number of protonation sites

**Fig. 3:**
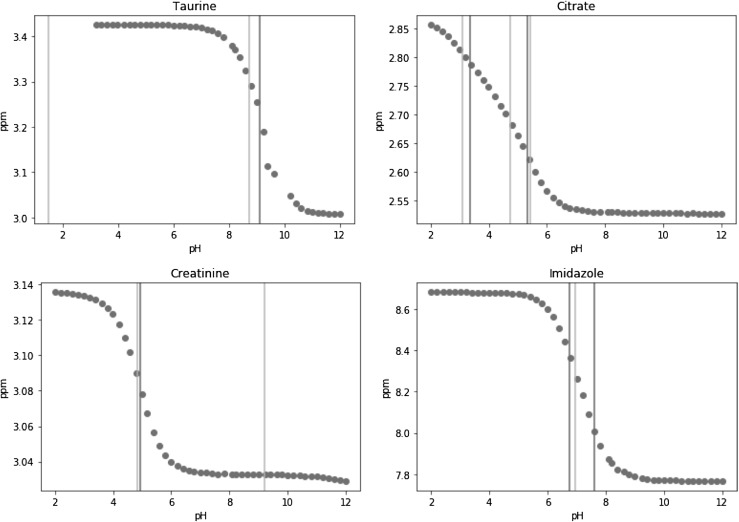
Examples of resonances with incorrectly estimated numbers of sites: taurine, citrate, creatinine, imidazole with literature pKa values (yellow line) and fitted pKa values (green line)

The model failed to estimate the correct number of protonation sites for 10 out of 51 resonances. There are several types of problem leading to incorrect estimation of the number of protonation sites. The first type ocurrs when at least one literature pKa value lies outside the range of the observed data. Taurine is a good example of this, as shown in Fig. 3, where it can be seen that one pKa lies at pH 1.5, while the data only cover the pH range 3.2–12.

The second type of inaccurate estimation happens when two adjacent pKas are so close that the change in chemical shift between them is too small compared to the measurement error. The $\delta$ 2.7 resonance of citrate is a good example of this, as in Fig. 3, where the smooth titration curve around pH 4–5 does not suggest the presence of the third pKa at 4.75. A third type of incorrect estmate happens when the change of chemical shift is too small so that the transition can not be detected near the pKa value, for instance creatinine as shown in Fig. 3. Conversely, the change in the chemical shift can be too large compared to the estimated measurement error, for example imidazole as shown in Fig. 3, forming a fourth type of inaccuracy.

Some molecules have multiple resonances and so the question arises of whether to combine them, or if not, how to pick the best resonance to model. We do not recommend to combine resonances from the same molecule as, with our data, this tended to over estimate the number of protonation sites leading to a poorer fit. Instead, it is preferred to pick a resonance with “good behaviour”, i.e. one which is not overlapped, shows strong changes in chemical shift, but with a good number of observations near each chemical shift transition (near the pKa). When more than one resonance from the same molecule are modelled and give different predictions for the number of sites, we recommend to use information such as the model fit error to judge which estimation is more reliable. We note that this does not apply in fully untargeted analysis when the metabolites are unidentified, and thus one does not know if two resonances come from the same molecule.

## Conclusions

The Bayesian fit based on the model of Szakács et al. (2004) can effectively estimate the number of protonation sites for many small molecule metabolites, given sufficient pH titration data. Incorrect estimations are mainly due to cases where pKa values are very similar, and thus could not be distinguished, and/or a lack of data in the necessary pH ranges. We note that, even when the number of sites was incorrectly estimated, it is still possible to estimate the chemical shift position of a resonance quite accurately in most cases. The information obtained from the modelling procedure described here could be useful in a number of ways. For example, the pH could be estimated from the positions of a few well known and easily located resonances. This could then be used to predict the chemical shift positions of resonances of other metabolites expected in a sample, which could then help with automated annotation, alignment or peak fitting (as an initial position estimate). The predicted number of protonation sites may also be helpful during the process of identifying unknown compounds, although orthogonal analytical information would almost always be needed in addition. Overall, we hope that this modelling approach may be valuable for the future development of algorithms for analysis of metabolomic $^1$ H NMR spectra including alignment, annotation and peak fitting.

## Data Availability

The metabolomics and metadata reported in this paper are available as supplementary information to the original study (Tredwell et al. 2016) which is available from the Springer website under the Creative Commons attribution 4.0 International License https://creativecommons.org/licenses/by/4.0/.

## References

[CR13] Ackerman JJH, Soto GE, Spees WM, Zhu Z, Evelhoch JL (1996). The NMR chemical shift pH measurement revisited: Analysis of error and modeling of a pH dependent reference. Magnetic Resonance in Medicine.

[CR1] Ebbels T, Cavill R (2009). Bioinformatic methods in NMR-based metabolic profiling. Progress in Nuclear Magnetic Resonance Spectroscopy.

[CR2] Fan TWM (1996). Metabolite profiling by one- and two-dimensional NMR analysis of complex mixtures. Progress in Nuclear Magnetic Resonance Spectroscopy.

[CR3] HMDB CA. (2017). Human metabolome database. http://www.hmdb.ca. Accessed 10 Oct 2017.

[CR4] Karakach T, Wentzell P, Walter J (2009). Characterization of the measurement error structure in 1D 1H NMR data for metabolomics studies. Analytica Chimica Acta.

[CR5] Lundblad R, Macdonald F (2010). Handbook of biochemistry and molecular biology.

[CR6] Plummer, M. (2003). JAGS: A program for analysis of Bayesian graphical models using Gibbs sampling. In *Proceedings of the 3rd international workshop on distributed statistical computing* (vol. 124).

[CR14] Szakács Z, Hägele G, Tyka R (2004). 1H/31P NMR pH indicator series to eliminate the glass electrode in NMR spectroscopic pKa determinations. Analytica Chimica Acta.

[CR7] Takis PG, SchÃd’fer H, Spraul M, Luchinat C (2017). Deconvoluting interrelationships between concentrations and chemical shifts in urine provides a powerful analysis tool. Nature Communications.

[CR8] Tredwell G, Bundy J, De Iorio M, Ebbels T (2016). Modelling the acid/base 1H NMR chemical shift limits of metabolites in human urine. Metabolomics.

[CR9] Vu T, Laukens K (2013). Getting your peaks in line: A review of alignment methods for NMR spectral data. Metabolites.

[CR10] Wishart DS, Jewison T, Guo AC, Wilson M, Knox C (2012). HMDB 3.0—The human metabolome database in 2013. Nucleic Acids Research.

[CR11] Wishart DS, Knox C, Guo AC (2009). HMDB: a knowledgebase for the human metabolome. Nucleic Acids Research.

[CR12] Wishart DS, Tzur D, Knox C (2007). HMDB: the human metabolome database. Nucleic Acids Research.

